# Reduction of fibroblast size/mechanical force down‐regulates TGF‐β type II receptor: implications for human skin aging

**DOI:** 10.1111/acel.12410

**Published:** 2015-10-08

**Authors:** Gary J. Fisher, Yuan Shao, Tianyuan He, Zhaoping Qin, Daniel Perry, John J. Voorhees, Taihao Quan

**Affiliations:** ^1^ Department of DermatologyUniversity of Michigan Medical SchoolAnn ArborMichigan

**Keywords:** aging, cell size, extracellular matrix, mechanotransduction, TGF‐β type II receptor, TGF‐β/Smad

## Abstract

The structural integrity of human skin is largely dependent on the quality of the dermal extracellular matrix (ECM), which is produced, organized, and maintained by dermal fibroblasts. Normally, fibroblasts attach to the ECM and thereby achieve stretched, elongated morphology. A prominent characteristic of dermal fibroblasts in aged skin is reduced size, with decreased elongation and a more rounded, collapsed morphology. Here, we show that reduced size of fibroblasts in mechanically unrestrained three‐dimensional collagen lattices coincides with reduced mechanical force, measured by atomic force microscopy. Reduced size/mechanical force specifically down‐regulates TGF‐β type II receptor (TβRII) and thus impairs TGF‐β/Smad signaling pathway. Both TβRII mRNA and protein were decreased, resulting in 90% loss of TGF‐β binding to fibroblasts. Down‐regulation of TβRII was associated with significantly decreased phosphorylation, DNA‐binding, and transcriptional activity of its key downstream effector Smad3 and reduced expression of Smad3‐regulated essential ECM components type I collagen, fibronectin, and connective tissue growth factor (CTGF/CCN2). Restoration of TβRII significantly increased TGF‐β induction of Smad3 phosphorylation and stimulated expression of ECM components. Reduced expression of TβRII and ECM components in response to reduced fibroblast size/mechanical force was fully reversed by restoring size/mechanical force. Reduced fibroblast size was associated with reduced expression of TβRII and diminished ECM production, in aged human skin. Taken together, these data reveal a novel mechanism that provides a molecular basis for loss of dermal ECM, with concomitant increased fragility, which is a prominent feature of human skin aging.

## Introduction

Human skin dermis is largely composed of a dense, collagen‐rich extracellular matrix (ECM), which provides structural and mechanical support for the skin (Uitto, 1986). The dermal ECM is primarily produced by fibroblasts, which in young skin attach to intact collagen fibrils through receptors on their surface. This attachment allows fibroblasts to generate contractile forces on the surrounding ECM. Mechanical resistance by the ECM promotes polymerization of the actin cytoskeleton and assembly of intracellular scaffolds that permit fibroblasts to spread.

During aging, the dermal ECM undergoes progressive fragmentation that impairs fibroblast attachment with consequent reduction of size (Varani *et al*., 2004; Fisher *et al*., 2008, 2009; Quan *et al*., 2013a). In aged skin, reduced size of fibroblasts is accompanied by their decreased production of key ECM components, such as type I collagen, fibronectin, and connective tissue growth factor (CTGF/CCN2) (Varani *et al*., 2004, 2006; Fisher *et al*., 2008; Quan *et al*., 2010).

Interestingly, many of the dermal ECM‐related genes that are down‐regulated in aged human skin are regulated by the TGF‐β pathway (Verrecchia & Mauviel, 2002; Quan *et al*., 2010). Importantly, components of the TGF‐β pathway itself are reduced in aged human skin (Quan *et al*., 2010), raising the possibility that impairment of TGF‐β signaling may be a major contributor to reduced ECM production in aged skin.

Dermal fibroblast size can be manipulated *in vitro* by culture on substrates of varying stiffness (Grinnell & Petroll, 2010; Janmey & Miller, 2011; Hopp *et al*., 2013; Buxboim *et al*., 2014). Fibroblasts achieve greater size on stiff substrates, compared to more compliant substrates. Reduced size is correlated with decreased production of ECM components (Arora *et al*., 1999; Kessler *et al*., 2001; Quan *et al*., 2013a). Although the relationship between fibroblast morphology and ECM production is well documented, the molecular basis of this relationship remains incompletely understood. Furthermore, there is little information about the relationship between the mechanical properties of fibroblasts and human skin connective tissue aging.

In this report, we quantify the mechanical properties of adult human dermal fibroblasts that have different size and investigate regulation of the TGF‐β pathway by fibroblast size/mechanical force. We find that reduced fibroblast size/mechanical force specifically down‐regulates TGF‐β type II receptor, and this down‐regulation largely mediates reduction of TGF‐β‐regulated ECM production. These data provide a foundation for understanding the cellular and molecular basis of age‐related decline of ECM production in human skin.

## Results

### Dermal fibroblasts cultured in unconstrained collagen matrix display reduced cell size and mechanical force

**Figure 1:**
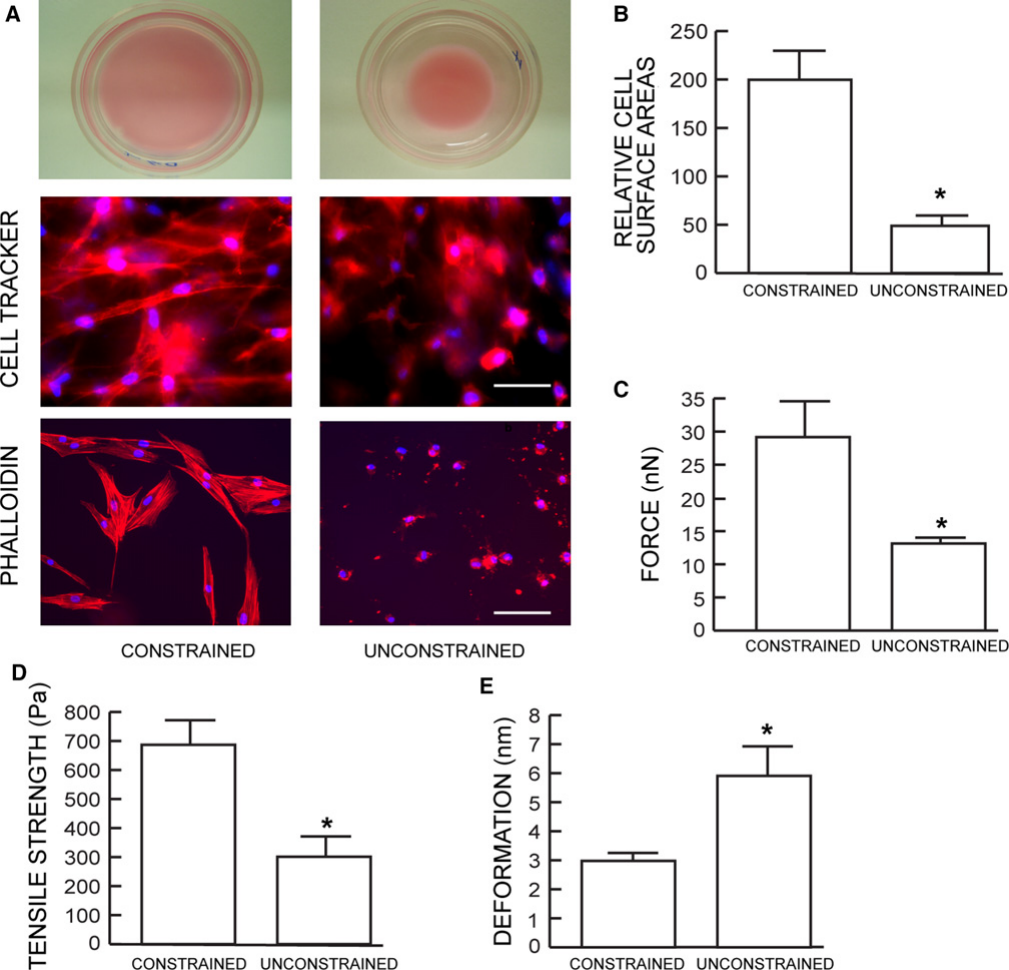
Dermal fibroblasts cultured in unconstrained collagen lattices display reduced size and mechanical force. (A) Dermal fibroblasts were cultured in mechanically constrained (upper left) or unconstrained (upper right) 3D type I collagen lattices. Cells were stained dye with CellTracker fluorescent, and nuclei were stained blue with DAPI (middle panels). Cellular actin filaments were stained red with phalloidin (lower panel) *N* = 8. (B) Relative cell surface areas were quantified by computerized image analysis (image‐pro plus software, version 4.1, Media Cybernetics, MD). Means ± SEM. *N* = 8, *P < 0.05. (C) traction forces, (D) tensile strength, and (E) deformation were determined by AFM PeakForce^™^ Quantitative NanoMechanics mode and nanoscope analysis software. Means ± SEM. *N* = 8, *P <* *0.05.

Primary adult human dermal fibroblasts were cultured in either mechanically constrained or unconstrained three‐dimensional collagen matrices. Mechanical constraint was achieved by incorporating a nylon mesh disk within the matrices (Kessler *et al*., 2001). The rigidity of the disk counter acted the traction force of the fibroblasts and thereby prevented contraction of the matrices (Fig. 1A top panels). Fibroblasts in restrained matrices displayed elongated, spread morphology, and prominent actin filaments (Fig. 1A middle and lower left panels). In contrast, fibroblasts in unconstrained matrixes displayed a rounded, contracted appearance, lacking distinct actin filaments (Fig. 1A middle and lower right panels). Quantitative morphometric analysis revealed that fibroblast surface area was reduced 76% in unconstrained matrices, compared to fibroblasts in constrained matrices (Fig. 1B).

We next employed atomic force microscopy (AFM) to determine changes in mechanical properties associated with reduced fibroblast size. The AFM probe tip was placed directly on the surface of the central regions of the fibroblasts. Key cellular mechanical properties, traction force (Fig. 1C) and tensile strength (Fig. 1D), were reduced 56% and 55%, respectively, while deformation was increased twofold (Fig. 1E), in unconstrained compared to constrained matrices.

### Reduced fibroblast size/mechanical force impairs TGF‐β signaling

**Figure 2:**
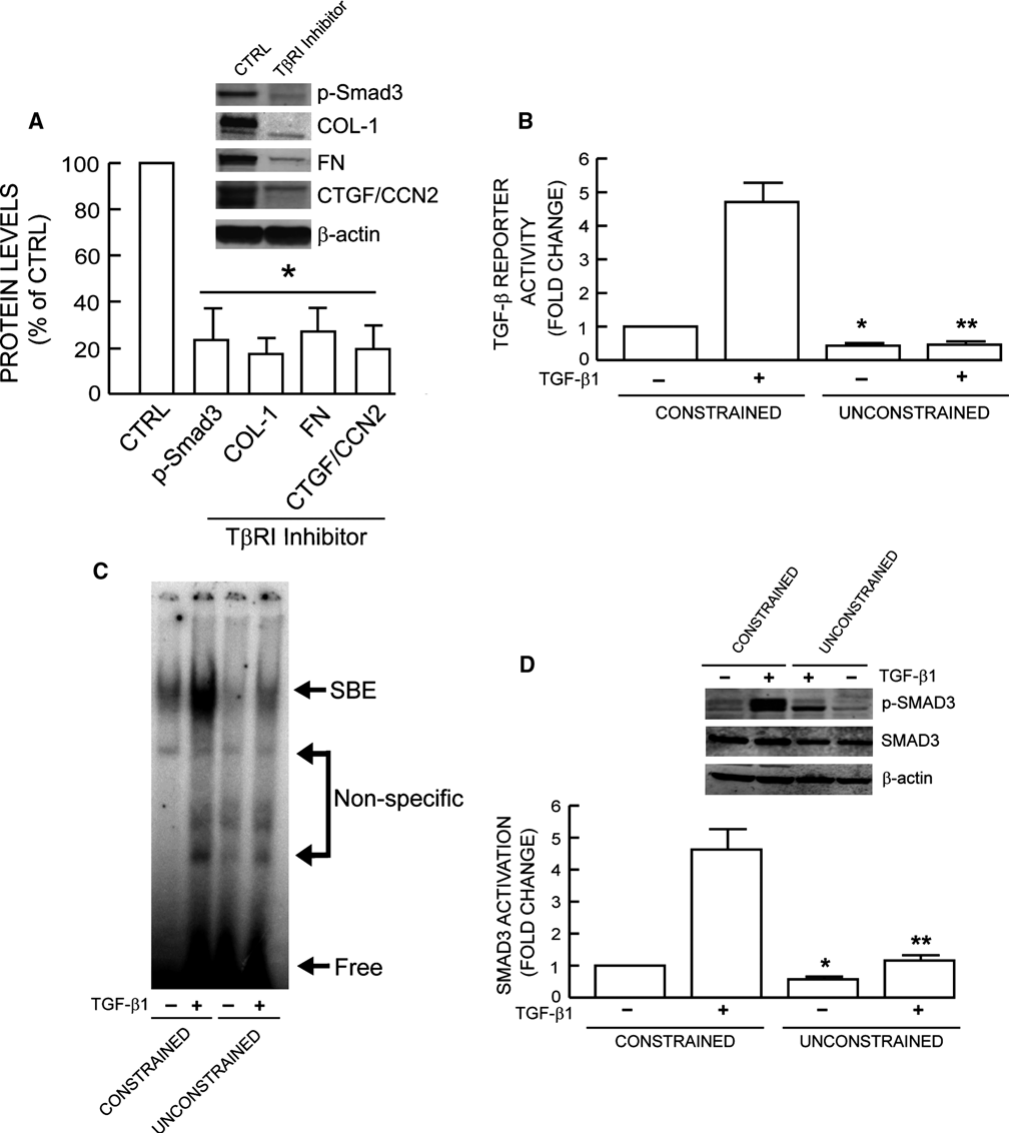
Reduced fibroblast size/mechanical force impairs TGF‐β signaling. (A) TGF‐β‐dependent ECM production. Constrained collagen lattices were treated with TβRI kinase inhibitor (SB431542, 10 μm) for 24 h. Smad3 phosphorylation and type I collagen, fibronectin, CTGF/CCN2 protein levels were determined by Western analysis and normalized to β‐actin (loading control). Inset shows representative Western blots. Mean ± SEM,* N* = 3, *P <* *0.05. (B) TGF‐β/Smad3 reporter activity. Cells were transfected with TGF‐β/Smad3‐dependent luciferase reporter construct (SBEX4). Twenty‐four hours after transfection, cells were harvested and cultured in collagen lattices for 1 day. Cultures were treated with TGF‐β1 (5 ng mL^−1^) for 16 h, and luciferase activity was determined. Mean±SEM,* N* = 3, *P <* *0.05 vs. control constrained, **P <* *0.05 vs. TGF‐β1 constrained. (C) Representative EMSA. Collagen lattice cultures were treated with TGF‐β1 (5 ng mL^−1^) for 4 h. Smad3 protein binding to target probe (containing the Smad3 binding element) was determined by EMSA,* N* = 3. (D) Smad3 phosphorylation. Collagen lattice cultures were treated with TGF‐β1 (5 ng mL^−1^) for 2 h. Smad3 phosphorylation was determined by Western analysis. Inset shows representative Western blots. Mean ± SEM,* N* = 3, *P <* *0.05 vs. control constrained, **P <* *0.05 vs. TGF‐β1 constrained.

Dermal fibroblast size/mechanical force is known to regulate production of key ECM components such as type I collagen, fibronectin, and connective tissue growth factor (CTGF/CCN2) (Kessler *et al*., 2001; Quan *et al*., 2010). As expected, we found that fibroblasts in unconstrained collagen matrices expressed reduced levels of these three genes (Fig. S1). As TGF‐β pathway is the major regulator of ECM production (Verrecchia & Mauviel, 2002; Quan *et al*., 2010), we confirmed that blocking of TGF‐β signaling by TGF‐β type I receptor kinase inhibitor (SB431542) resulted in significant reduction of Smad3 phosphorylation, type I collagen, fibronectin, and CTGF/CCN2 in constrained cultures (Fig. 2A). We next assessed TGF‐β signaling using a TGF‐β/Smad3‐dependent luciferase reporter construct (SBEX4) (Quan *et al*., 2001). Both basal and TGF‐β‐induced reporter activities were markedly reduced in fibroblasts with reduced size/mechanical force (Fig. 2B). Consistent with these results, both basal and TGF‐β‐induced binding of Smad3 to its DNA response element were substantially inhibited by reduced fibroblast size/mechanical force (Fig. 2C). Furthermore, TGF‐β‐dependent Smad3 phosphorylation, which is required for transcriptional activity, was significantly inhibited by reduced size/mechanical force. No change in the level of total Smad3 protein was observed (Fig. 2D).

### Reduced fibroblast size/mechanical force specifically down‐regulates TβRII

**Figure 3:**
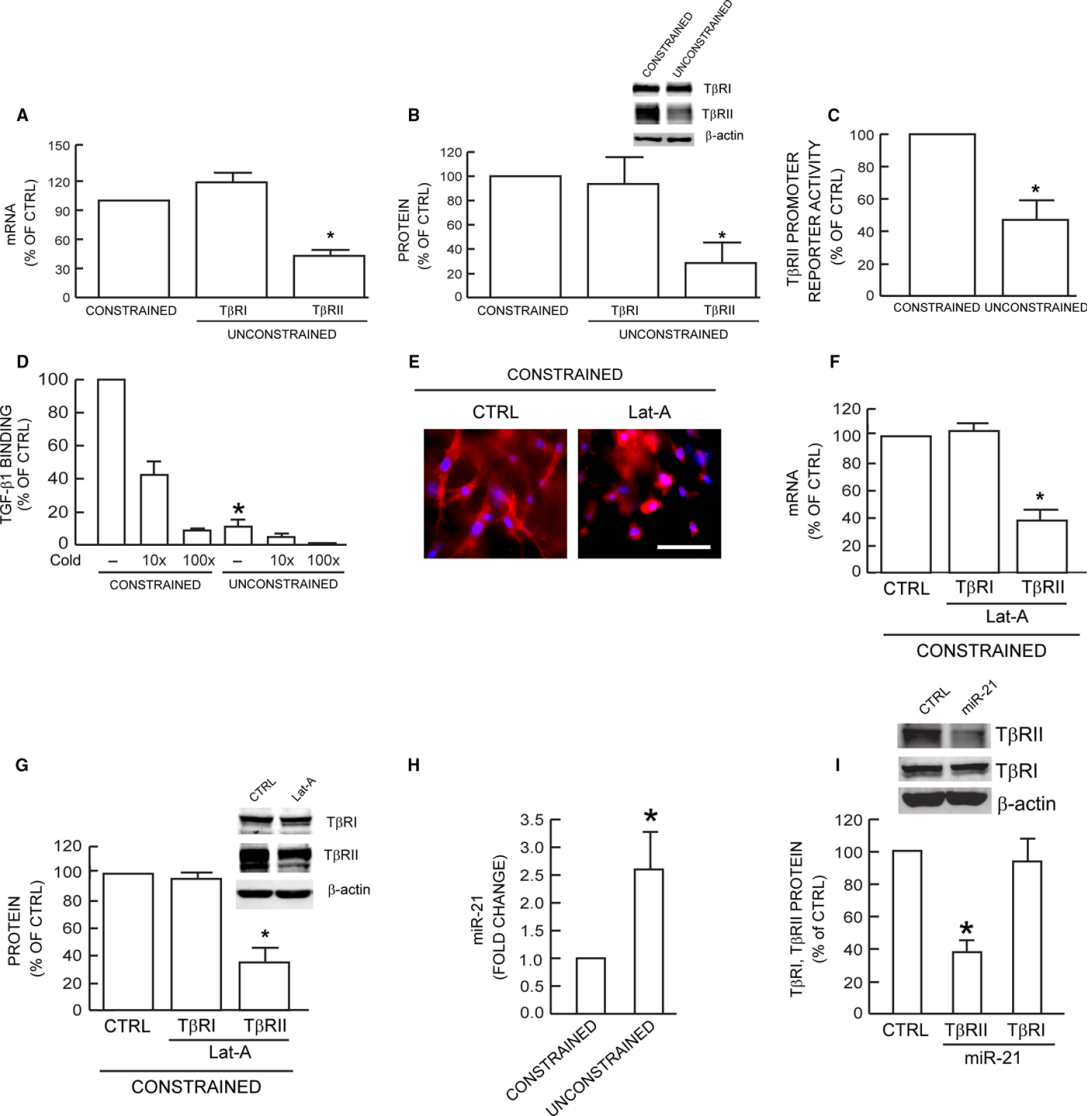
Reduced fibroblast size/mechanical force specifically down‐regulates TβRII. TβRI and TβRII (A) mRNA and (B) protein levels were determined by real‐time RT–PCR and Western analysis, respectively. mRNA and protein levels were normalized to 36B4 (internal housekeeping gene control) and β‐actin (loading control), respectively. Inset shows representative Western blots. Mean ± SEM,* N* = 8, *P <* *0.05. (C) TβRII promoter reporter activity. Fibroblasts were transfected with TβRII promoter reporter. Twenty‐four hours transfection, cells were harvested and cultured in collagen lattices for two days, prior to measurement of luciferase activity. Mean ± SEM,* N* = 3, *P <* *0.05. (D) Specific binding of [^125^I]TGF‐β1 to fibroblasts. Specificity of TGF‐β1 binding to receptor complex was confirmed by competition assay by addition of 10–100× excess of unlabeled TGF‐β1. Means ± SEM,* N* = 3, *P <* *0.05. (E) Fibroblasts were cultured in type I collagen lattices and treated with DMSO (CTRL, left) or Latrunculin‐A (Lat‐A, right, 100 nm) for 24 h. Fibroblasts were stained with CellTracker red fluorescent dye. TβRI and TβRII (F) mRNA and (G) protein levels were quantified by real‐time RT–PCR and Western analysis, respectively. mRNA and protein levels were normalized by 36B4 (housekeeping gene internal control) and β‐actin (loading control), respectively. Inset shows representative Western blots. Mean ± SEM,* N* = 3, *P <* *0.05. (H) miR‐21 levels were determined by real‐time PCR and normalized to RNU6B, endogenous reference snRNA. Mean ± SEM,* N* = 3, *P <* *0.05. (I) Fibroblasts were treated with miR‐21 mimic for 24 h. Protein levels were determined by Western analysis and normalized to β‐actin (loading control). Inset shows representative Western blots. Mean ± SEM,* N* = 3, *P <* *0.05.

The above data suggest that reduced size/mechanical force interferes with the ability of the TGF‐β receptor complex, composed of TβRI and TβRII, to phosphorylate Smad3. To investigate this possibility, we next examined the effect of fibroblast size/mechanical force on TβRI and TβRII gene expression. We found that reduced size/mechanical force did not alter TβRI mRNA or protein levels (Fig. 3A,B). Other TGF‐β pathway components such as TGF‐β ligands and Smads also remained unchanged (Fig. S2). In contrast, TβRII mRNA (Fig. 3A) and protein (Fig. 3B) levels were significantly decreased by 57% and 72%, respectively. Reduced size/mechanical force also significantly reduced TβRII promoter activity (Fig. 3C), suggesting reduced TβRII mRNA and protein results, at least in part, from decreased gene transcription. TβRII is necessary for TGF‐β ligand binding to TGF‐β receptor complex, the initial step of TGF‐β signaling. As shown in Fig. 3D, TGF‐β1‐binding to fibroblasts was reduced approximately 90% by reduced size/mechanical force. Specificity of TGF‐β1‐binding was confirmed by competition with unlabeled TGF‐β1. To further investigate the connection between reduced size/mechanical force and specific down‐regulation of TβRII, we treated fibroblasts in constrained collagen matrices with latrunculin‐A (Lat‐A), which rapidly blocks actin polymerization (Gieni & Hendzel, 2008). As expected, disruption of the actin cytoskeleton resulted in reduced fibroblast size (Fig. 3E). In these fibroblasts, gene expression of TβRII, but not TβRI, was specifically down‐regulated (Fig. 3F,G). These data suggest that reduced fibroblast size/mechanical force results in specific down‐regulation of TβRII.

In addition to transcriptional regulation, TβRII is regulated by post‐transcriptional mechanisms. Recently, it has been reported that microRNA‐21 (miR‐21) reduces TβRII expression through direct interaction with 3' nontranslated sequences in the TβRII transcript (Kim *et al*., 2009; Yu *et al*., 2012). Therefore, we next explored the potential role of miR‐21 in down‐regulation of TβRII expression in response to the reduced fibroblast size/mechanical force. Interestingly, we found that miR‐21 was significantly induced 2.5‐fold by reduced size/mechanical force (Fig. 3H). Furthermore, addition of miR‐21 mimic resulted in down‐regulation of TβRII, but not TβRI expression (Fig. 3I). These data indicate that the level of miR‐21 is responsive to size/mechanical force and may participate in the mechanism of down‐regulation of TβRII expression.

### Expression of TβRII restores TGF‐β signaling and induction of ECM components

**Figure 4:**
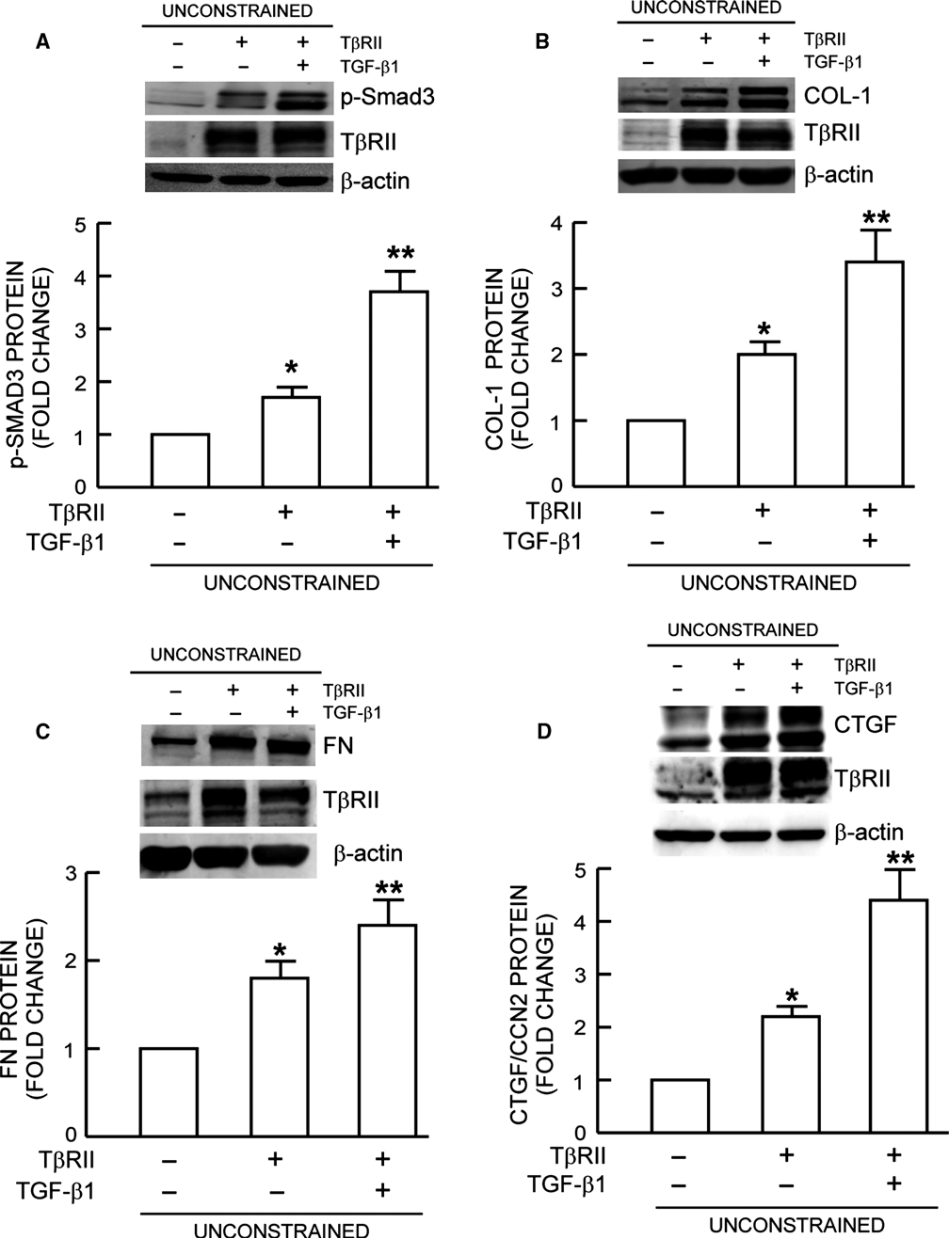
Expression of TβRII restores TGF‐β signaling and induction of ECM components. Fibroblasts were infected with control adenovirus virus or TβRII adenovirus. Twenty‐four hours after infection, cells were harvested and cultured in mechanically unconstrained collagen lattices for 1 day. Cultures were treated with TGF‐β1 (5 ng mL^−1^) for (A) one hour or (B, C, and D) 16 h. (A) Smad3 phosphorylation, (B) Type I procollagen, (C) Fibronectin, and (D) CTGF/CCN2 protein levels were determined by Western analysis. Protein levels were normalized by β‐actin (loading control). Insets show representative Western blots. Mean ± SEM,* N* = 3 for (A), *N* = 4 for (B, C, and D), *P <* *0.05 vs. control, **P <* *0.05 vs. no TGF‐β1.

The above data suggest that down‐regulation of TβRII may mediate impairment of TGF‐β signaling and decreased expression of ECM components in response to reduced size/mechanical force. To investigate this possibility, we expressed TβRII in fibroblasts in unconstrained matrices with reduced size/mechanical force. Restoration of TβRII expression did not change the fibroblasts morphology (data not shown). However, compared to fibroblasts transfected with control vector, expression of TβRII increased basal and TGF‐β1‐induced Smad3 phosphorylation fourfold (Fig. 4A). Restoration of TβRII also elevated basal and TGF‐β1‐induced protein levels of type I procollagen (Fig. 4B), fibronectin (Fig. 4C), and CTGF/CCN2 (Fig. 4D). These data demonstrate that down‐regulation of TβRII is key event that mediates impaired TGF‐β signaling and consequently decreased ECM production in response to reduced fibroblast size/mechanical force.

### Restoration of fibroblast size/mechanical force reverses impaired TGF‐β signaling and ECM component production

**Figure 5:**
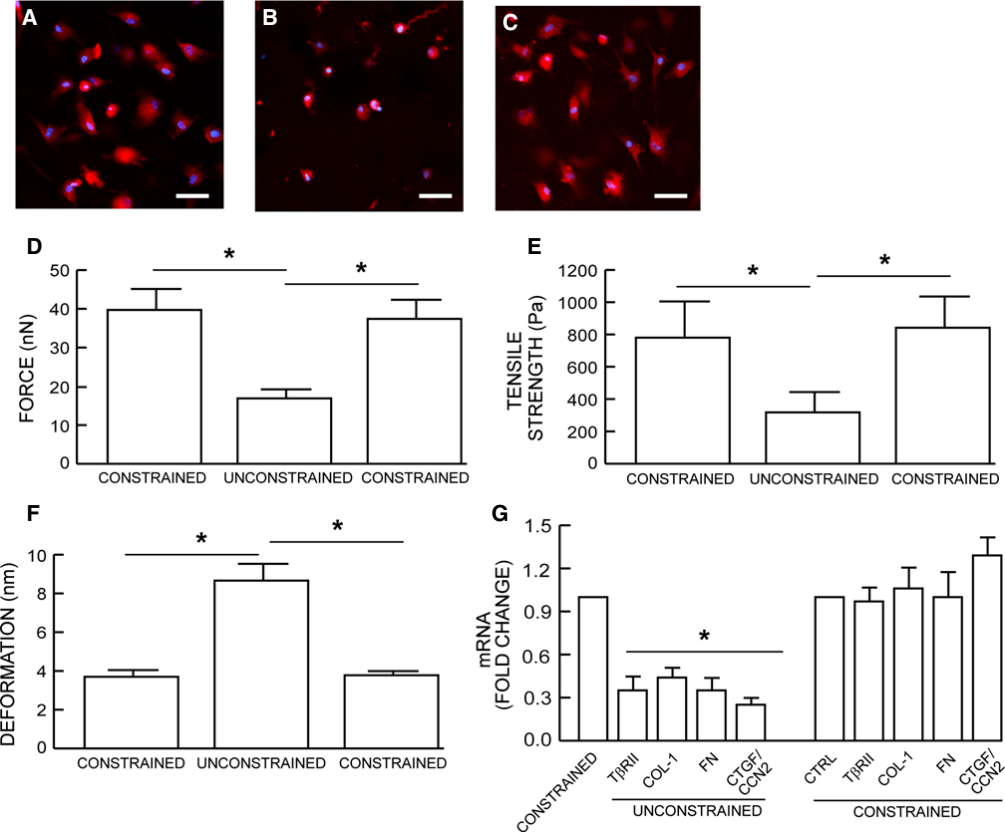
Restoration of fibroblast size/mechanical force reverses impaired TGF‐β signaling and ECM component production. Fibroblasts were cultured in mechanically (A) constrained or (B) unconstrained 3D type I collagen lattices. Fibroblasts were harvested from mechanically (B) unconstrained collagen lattices and cultured in mechanically (C) constrained collagen lattices. Cell morphology and nuclei were visualized by staining cells with CellTracker red fluorescent dye, and DAPI (blue), respectively. Cell mechanical properties, (D) traction forces and (E) tensile strength, and (F) deformation, were determined by AFM PeakForce^™^ Quantitative NanoMechanics mode and nanoscope analysis software, as described in *Method*. Means ± SEM. *N* = 8, *P <* *0.05. (G) Type I procollagen, fibronectin, and CTGF/CCN2 mRNA levels were determined by real‐time RT–PCR. mRNA levels were normalized by the housekeeping gene (36B4, internal control). Mean ± SEM,* N* = 4, *P <* *0.05.

We assessed the reversibility of TβRII‐dependent alterations that occur due to reduced size/mechanical force. Small, rounded fibroblasts were harvested from unconstrained collagen matrices (Fig. 5B) and placed into constrained matrices (Fig. 5C). In this constrained environment, the fibroblasts displayed spread morphology (Fig. 5C), indistinguishable from fibroblasts maintained in constrained matrices (Fig. 5A). Transfer to constrained matrices also fully restored fibroblast mechanical properties. Traction force (Fig. 5D) and tensile strength (Fig. 5E) were increased, and deformability (Fig. 5F) was decreased to levels similar to fibroblasts in constrained matrices. Consistent with the recovery of fibroblast size/mechanical force, reduced expression of TβRII, type I collagen, fibronectin, and CTGF/CCN2 was restored to levels of fibroblasts in constrained matrices (Fig. 5G).

### Reduced TβRII and ECM gene expression in aged human skin

**Figure 6:**
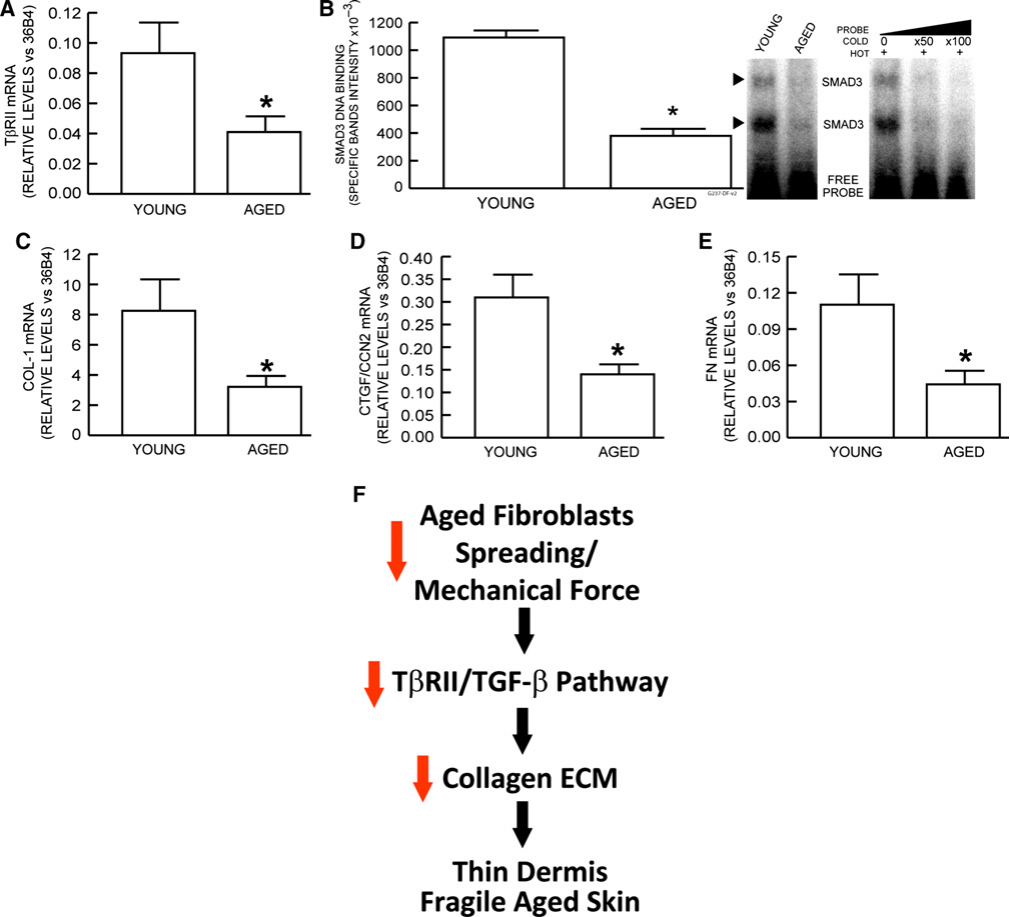
Reduced gene expression of TβRII and ECM components in aged human skin. Dermal fibroblasts from buttocks skin of young (<21–30 years old) and aged (>80 years old) were obtained by laser capture microdissection. mRNA levels of indicated genes were quantified by real‐time RT–PCR. mRNA levels were normalized to internal control, housekeeping gene 36B4. (A) type II TGF‐β receptor, (B) Nuclear extracts were prepared from young and aged human skin dermis. Smad3 protein binding to target probe, containing consensus Smad3 binding element, was determined by EMSA. Specificity of retarded complexes was determined by addition of excess unlabeled probe. Intensities of specific retarded complex bands were quantified by Storm Molecular Imager. Means ± SEM,* N* = 6, *P <* *0.05. (C) type I procollagen, (D) connective tissue growth factor/CCN2, (E) fibronectin. Means ± SEM,* N* = 12, *P <* *0.05. (F) Reduced fibroblast size/mechanical force contributes to skin aging by impairment of TGF‐β signaling and inhibition of ECM production. See details in Discussion.

We previously reported that fibroblasts in aged human skin display a contracted, rounded morphology with 50% less surface area (Varani *et al*., 2006). Therefore, we determined whether reduced fibroblast size in aged skin is associated with reduced TβRII gene expression. Dermal fibroblasts were obtained from young (21–30 years old) and aged (>80 years old) human buttocks skin by laser capture microdissection. TβRII gene expression was reduced nearly 60% in aged, compared to young skin (Fig. 6A). This reduction was associated with decreased activation of TβRII downstream effector Smad3, measured by electrophoretic mobility shift assay, using nuclear extracts from young and aged dermis (Fig. 6B). Consistent with these results, type I procollagen (Fig. 6C), CTGF/CCN2 (Fig. 6D), and fibronectin (Fig. 6E) gene expression were also significantly reduced in aged human skin.

## Discussion

Recent studies highlight the importance of physical forces within cells and tissues in the regulation of key biological processes including growth, differentiation, development, and cancer (Discher *et al*., 2005; Assoian & Klein, 2008; Butcher *et al*., 2009; Janmey & Miller, 2011; Janmey *et al*., 2013; Humphrey *et al*., 2014; Iskratsch *et al*., 2014). The impact of mechanobiology on cutaneous biology and in particular skin aging is relatively unexplored. In this report, we used a three‐dimensional collagen matrix model to investigate the relationships among dermal fibroblast morphology, mechanical forces, and expression of ECM components.

In human dermis, fibroblasts are embedded in a collagen‐rich microenvironment and physically interact with collagen fibrils to maintain normal cell size and mechanical force. In constrained matrices, traction forces are counterbalanced by mechanical resistance of the collagen matrix. These conditions establish mechanical tension, which promotes spread morphology, and production of ECM components. In unconstrained matrices, traction forces meet little resistance, and the cells and the matrix contract. Under these conditions, mechanical tension and production of ECM components are reduced. In constrained matrices, reducing fibroblast size/mechanical force by treatment with latrunculin‐A, which causes depolymerization of the actin cytoskeleton, also results in a decline of ECM component production. Thus, regulation of ECM production is not dependent on collagen matrix contraction *per se*, but rather reflects the mechanical state of the fibroblasts.

Recent studies have begun to unravel mechanisms by which mechanical forces are sensed and converted to cellular responses (Janmey & Miller, 2011; Janmey *et al*., 2013; Mammoto *et al*., 2013). For example, it has been shown that traction forces exerted by cells on the ECM expose cryptic domains within collagen and fibronectin that can stimulate intracellular signaling pathways affecting cellular growth and ECM turnover (Hocking & Kowalski, 2002; Vogel & Sheetz, 2009; Orgel *et al*., 2011; Bonnans *et al*., 2014). The actin myosin cytoskeleton is the primary source of force generation by cells. Recent studies have elucidated two independent signal transduction pathways that are regulated by the state of actin polymerization. The effectors of these pathways are transcription factors YAP and MRTF (Olson & Nordheim, 2010; Dupont *et al*., 2011; Janmey *et al*., 2013; Maller *et al*., 2013). Both transcription factors are sequestered in the cytoplasm when bound to separate protein complexes that associate with monomeric G‐actin. Mechanical force generation through recruitment of G‐actin to actin filaments results in release of YAP or MRTF, which translocate to the nucleus to regulate expression of target gene (Olson & Nordheim, 2010; Dupont *et al*., 2011; Maller *et al*., 2013).

In this report, we identify a novel mechanism by which reduced fibroblast size/mechanical force impairs TGF‐β/Smad signaling by down‐regulation of TGF‐β type II receptor. The TGF‐β pathway is well established as a major regulator of ECM production (Varga, 2002; Verrecchia & Mauviel, 2002; Pohlers *et al*., 2009; Quan *et al*., 2010). It is regulated by a variety of mechanisms including ligand processing, phosphorylation, and inhibitory Smad proteins (Massague, 2012). ECM mechanical remodeling has been shown to regulate TGF‐β1 activation by releasing TGF‐β1 from the ECM microenvironment (Wells & Discher, 2008; Klingberg *et al*., 2014). Our findings demonstrate another level of regulation of TGF‐β by mechanical force; reduced mechanical force can down‐regulate the ability of cells to respond to TGF‐β via specific down‐regulation of TβRII. Down‐regulation of TβRII decreases TGF‐β‐binding, which is required for TβRI activation and consequent Smad3 phosphorylation. Neither TβRI nor Smad3 levels are altered; however, Smad3 phosphorylation, DNA‐binding, and transcriptional activity are reduced. Reduced Smad3 activity suppresses expression of target genes that include key components of the ECM.

Reduced fibroblast size/mechanical force decreases the levels of both TβRII mRNA and protein. TβRII promoter activity is also reduced, suggesting that down‐regulation of TβRII expression may result, at least in part, from decreased gene transcription. Obviously, it is of interest to identify *trans* and *cis*‐acting factors that mediate size/mechanical force‐dependent regulation of TβRII gene transcription. Neither YAP nor MRTF, which are regulated by mechanical force described above, or TAZ, which is closely related to YAP appear to be involved in regulation of TβRII in dermal fibroblasts (TQ and GF unpublished data). It is conceivable that post‐transcriptional mechanisms may also contribute to regulation of TβRII protein levels by size/mechanical force. Reduced TβRII protein could result from reduced synthesis and/or increased degradation. Interestingly, we found that miR‐21, which has been shown to inhibit TβRII protein synthesis (Kim *et al*., 2009; Yu *et al*., 2012), is significantly induced by reduced fibroblast size/mechanical force. These data suggest a potential mechanism in which miR‐21 functions as a mechanosensitive microRNA to mediate down‐regulation of TβRII expression. Clearly, additional studies are warranted to uncover the precise molecular mechanism(s) by which reduced fibroblast size/mechanical force down‐regulates TβRII expression.

The dermal ECM becomes progressively fragmented during aging. Fibroblasts are unable to attach to the fragmented ECM and therefore cannot exert traction forces needed to spread, and appear contracted. We and others have previously reported that many TGF‐β‐regulated genes involved in ECM production are reduced in aged human skin (Varani *et al*., 2006; Quan *et al*., 2010). Importantly, TβRII expression is also reduced in aged skin (Quan *et al*., 2006; Quan & Fisher, 2015), suggesting that impaired TGF‐β signaling may be a key mediator of reduced ECM production. This possibility is supported by recent evidence demonstrating that ECM production can be significantly stimulated in aged human skin by enhancing structural support of the dermis with injectable cross‐linked hyaluronic acid dermal filler (Quan *et al*., 2013b). Localized increase in mechanical force causes fibroblasts in proximity to the filer to enlarge and up‐regulate TβRII expression. These enlarged fibroblasts produce increased levels of collagen and CTGF/CCN2. Interestingly, we recently reported that reduced fibroblast size/mechanical force also up‐regulates matrix metalloproteinase‐1 expression and thus causes collagen fibril fragmentation (Qin *et al*., 2014), as observed in aged human skin *in vivo* (Fisher *et al*., 2008, 2009). These data combined with our current findings provide a foundation for our understanding the cellular and molecular basis of age‐related loss of collagen production and increased collagen fragmentation in aged human skin.

We propose a working model of human dermal aging (Fig. 6F). Age‐associated ECM fragmentation reduces dermal fibroblast size/mechanical force, leading to impairment of TGF‐β signaling, via specific down‐regulation of TβRII. Decreased TβRII in turn leads to decline of dermal fibroblast ECM production and thus contributes to skin thinning, with attendant fragility, diminished wound healing, and functional impairment of blood vessels and appendages.

## Experimental procedures

### Materials

Dulbecco's Modified Eagle's Media (DMEM), fetal calf sera, trypsin solution, and penicillin/streptomycin were obtained from Invitrogen Life Technology (Carlsbad, CA, USA). [γ‐^32^P]ATP was obtained from New England Nuclear Life Science Products (Boston, MA, USA). Rat tail type I collagen was purchased from BD Biosciences (Palo Alto, CA, USA). TGF‐β1 was purchased from R&D Systems (Minneapolis, MN, USA). Latrunculin‐A was purchased from Enzo Life Sciences (Farmingdale, NY, USA). All other reagents were purchased from Sigma Chemical Company (St. Louis, MO, USA).

### Procurement of human skin samples and laser capture microdissection (LCM)

Sun‐protected buttock human skin punch biopsies were obtained from clinically normal adult volunteers, 21–30 years for young group (*N* = 12, mean age 26 ± 3 years) males and 80+ years for aged group (*N* = 12, mean age 83 ± 4 years). Skin samples were 4 mm in diameter, full thickness skin. As previously described (Quan *et al*., 2013b), approximately 200 fibroblasts from each cryosection (15 μm) were collected in lysis buffer (RNeasy Micro kit, Qiagen, Chatsworth, CA, USA), followed by RNA extraction and RT–PCR, as described above. All procedures involving human subjects were approved by the University of Michigan Institutional Review Board, and all subjects provided written informed consent.

### Human dermal fibroblasts culture in 3D collagen lattices

Primary adult human dermal fibroblasts were prepared from 4‐mm full‐thickness punch biopsies of healthy volunteers, as previously described (Fisher *et al*., 1991). Cells between passage 3 and 10 were used for all experiments. 3D collagen lattices were prepared based on previous publication with minor modification (Fisher *et al*., 2009). Briefly, neutralized rat tail type I collagen (2 mg mL^−1^, BD, Biosciences) was suspended in medium cocktail [DMEM, NaHCO_3_ (44 mm), L‐glutamine (4 mm), Folic Acid (9 mm), and neutralized with 1N NaOH to pH 7.2]. 1 × 10^6^ cells were suspended in 2 mL collagen and medium cocktail solution and plated in a 35‐mm bacterial culture dish. These collagen lattices were placed in an incubator at 37 °C for 30 min to allow collagen polymerization. The collagen lattices were then incubated with 2 mL media (DMEM, 10% FBS) at 37 °C, 5% CO_2_. For the tensioned culture system, cells were cultured in collagen gel in which a nylon mesh (0.5 mm pore size) was placed on the bottom of culture dish to provide cytoskeletal stability and tension. For the relaxed culture system, dermal fibroblasts were cultured in collagen lattices without nylon mesh. For analyses, collagen lattices were either embedded in OCT for cryostat sectioning, or fibroblasts were harvested by digesting collagen gel with bacterial collagenase (1 mg mL^−1^, Sigma) for 30 min at 37 °C. Fibroblasts were collected by centrifugation, and recovery of viable fibroblasts (>80%) was confirmed by trypan blue staining. Where indicated, cultures were treated with TGF‐β1 (5 ng mL^−1^) for indicated times.

### RNA isolation and quantitative real‐time RT–PCR

RNA isolation and quantitative real‐time RT–PCR were performed as described previously (Quan *et al*., 2001). Briefly, cells were isolated from the collagen gel as described above, and total RNA was extracted with a commercial kit (RNeasy mini kit, Qiagen) according to the manufacturer's protocol. A total of 100 ng total RNA was reverse transcribed using Taqman Reverse Transcription kit (Applied Biosystems, Foster City, CA, USA). Real‐time RT–PCR was performed using a Taqman Universal PCR Master Mix kit (Applied Biosystems) and 7300 Sequence Detector (Applied Biosystems). Col‐1, FN, CTGF/CCN2, and TβRII primers and probes were purchased from Applied Biosystems (Assays‐on‐Demand^™^ Gene Expression Products). Target gene mRNA levels were normalized to the housekeeping gene 36B4 (a ribosomal protein used as an internal control for quantitation) levels as an internal control. miR‐21 levels were determined by real‐time PCR using TaqMan^®^ MicroRNA Assays Kit (Life Technology, NY, USA) according to the manufacture's protocol. miR‐21 mimic was purchased from Exiqon, Inc (Woburn, MA, USA).

### Western blots

Western blots were performed as described previously (Quan *et al*., 2001). Briefly, cells were isolated from collagen lattices, and whole cell extract was prepared by suspension of cells with whole cell extraction buffer (25 mm HEPES [pH 7.7], 0.3 m NaCl, 1.5 mm MgCl_2_, 0.2 mm EDTA, 0.1% Triton X‐100, 0.5 mm DTT, 20 mm β‐glycerolphosphate, 0.1 mm Na_3_VO_4_, 2 μg mL^−1^ leupeptin, and 100 μg mL^−1^ PMSF) followed by centrifugation. Concentration of proteins was determined by Bio‐Rad protein assay (Bio‐Rad laboratories, Hercules, CA, USA) using bovine serum albumin as a standard. Whole cell extract (20–50 μg) was resolved on 10% SDS‐PAGE, transferred to PVDF membrane, and blocked with PBST (0.1% Tween 20 in PBS) containing 5% milk for one hour at room temperature. The primary antibodies, Col‐1 (Southern Biotech, Birmingham, AL, USA), FN (SC‐18827, P5F3, Lot #:E0912, Santa Cruz Biotechnology, Santa Cruz, CA, USA), CTGF/CCN2 (SC‐14939, L20, Lot #: B1214, Santa Cruz Biotechnology 214), TβRI (SC‐398, V‐22, Lot #: H1310, Santa Cruz Biotechnology), TβRII (SC‐400, L‐21, Lot #: A1516, Santa Cruz Biotechnology), and phospho‐Smad3 (Cell Signaling Technology, Danvers, MA, USA), were incubated with PVDF membrane for one hours at room temperature. Blots were washed three times with PBST solution and incubated with appropriate secondary antibody for one hour at room temperature. After washing three times with PBST, the blots were developed with ECF (Vistra ECF Western Blotting System, Amersham Pharmacia Biotech, Piscataway, NJ, USA) following the manufacturer's protocol (Molecular Dynamics, Sunnyvale, CA, USA). The blots were scanned by STORM PhosphorImager, and intensities of each band were normalized with β‐actin (Sigma) as loading control. The specificity of the TβRII and TβRI antibodies was confirmed by knockdown TβRII and TβRI siRNAs, respectively (Fig. S3). TβRII (AACGGTGCAGTCAAGTTTCCA) and TβRI (AATTCCTCGAGATAGGCCGTT) siRNAs were purchased from Qiagen.

### Transient transfection and luciferase reporter assays

Primary adult human dermal fibroblasts were transiently transfected with TβRII promoter/reporter construct and TGF‐β/Smad3‐dependent luciferase reporter construct (SBEX4) (Quan *et al*., 2001) by electroporation using human dermal fibroblasts nucleofector kit (Amaxa Biosystems, Gaithersburg, MD, USA). β‐galactosidase expression vector was used as an internal control for transfection efficiency. After 24 h incubation, the transfected cells were cultured in 3D collagen lattices for 48 h, as described above. The cells were collected by digesting collagen with bacterial collagenase, and aliquots containing identical β‐galactosidase activity were used for each luciferase assay. Luciferase activity was measured using an enhanced luciferase assay kit (PharMingen International, San Diego, CA, USA) according to the manufacturer's protocol.

### Electrophoretic mobility shift assay (EMSA)

Electrophoretic mobility shift assay was performed as described previously (Qin *et al*., 2014). Briefly, nuclear extracts prepared using Nuclear and Cytoplasmic Extraction reagents (Pierce, Rockford. IL, USA). Double‐stranded oligodeoxynucleotides containing the Smad binding element (SBE) (5'‐TCGAGAGCCAGACAAGGAGCCAGACAAGGAGCC‐AGACAC‐3' and its complementary strand) were used as probe. All oligonucleotides were synthesized from Invitrogen (Grand Island, NY, USA). The probe was 5'‐end‐labeled with [γ‐^32^P]ATP using T4 polynucleotide kinase (Life Technologies Inc., Grand Island, NY, USA). The end‐labeled probe was purified with a G50 column (Roche Molecular Biochemicals, Indianapolis, IN, USA). Approximately 2 × 10^5^ cpm of end‐labeled DNA probe was incubated with 5 μg of nuclear extract on ice for 30 min. Protein‐DNA complexes were electrophoresed on 4% polyacrylamide gel at 30 mA for 100 min in TBE running buffer (1.0 m Tris, 0.9 m boric acid, 0.01 m EDTA). The gel was transferred to Whatman paper, vacuum‐dried, and scanned by STORM PhosphorImager.

### Adeno‐X expression vector construction and infection

TβRII cDNA was amplified by PCR, and gel‐purified PCR product was subcloned into a TA cloning vector (pCR2.1, Invitrogen, San Diego, CA). Purified pCR2.1 DNA was digested with restriction enzymes, and the excised inserts were gel purified and ligated into pShuttle. TβRII/pShuttle was used to generate the Adeno‐X expression vector using the Adeno‐X expression system (Clontech Laboratories, Inc., Temecula, CA, USA) according to the manufacturer's protocol. All constructs were subjected to restriction enzyme digestion analysis and nucleotide sequencing to verify correct sequence and orientation. Adenovirus was amplified by infection of HEK‐293FT cells and purified by sucrose gradient centrifugation. Cells were infected with control adenoviruses and TβRII adenovirus at a multiplicity of infection of 100 plaque‐forming units/cell. Adenovirus‐infected cells were harvested at 24 h postinfection and embedded in collagen gel, as described above.

### Whole Cell Binding of ^125^I‐ TGF‐β1

Binding of ^125^I‐ TGF‐β1 to cells was performed as previously described with minor modifications (Quan *et al*., 2001). Briefly, collagen lattices were washed three times with Krebs‐Ringer‐Hepes/BSA binding buffer (50 mm Hepes, pH 7.5, 128 mm NaCl, 1.3 mm CaCl_2_, 5 mm KCl, 0.5% BSA) and then incubated in Krebs‐Ringer‐Hepes/BSA for 30 min at 37 °C, 5% CO_2_. Collagen lattices were washed with 0.1% glacial acetic acid for 5 min at room temperature, placed in Krebs‐Ringer‐Hepes/BSA, and then 0.2 nm ^125^I‐ TGF‐β1 was added for 4 h at 4 °C. Nonspecific binding was determined by addition of 10× to 20× excess of unlabeled TGF‐β1. Cells were harvested as described above, and radioactivity was measured by scintillation counting, as described previously (Quan *et al*., 2001).

### Immunohistology, CellTracker, and phalloidin staining

Immunohistology was performed as described previously (Quan *et al*., 2010). Briefly, cryo‐sections (7 μm) were fixed in 5% paraformaldehyde for two hours at room temperature and were incubated with 0.5% Nonidet P‐40 and then blocked with 2% bovine serum albumin (BSA). The slides were washed with PBS five times and incubated with Col‐1 primary antibody (Santa Cruz Biotechnology) for 1 h at room temperature, followed by incubation with Super Sensitive MultiLink (BioGenex, Fremont CA, USA) for 10 min and SS Label (BioGenex) for 10 min. Then, the slides were developed with One Step AEC Soln (BioGenex) for 3 min and counterstained with hematoxylin (BioCare, Concord, CA, USA) for 20 s and mounted with Supermount (BioGenex). Control staining was performed with corresponding rabbit immunoglobulin and confirmed no immunoreactivity (data not shown). Cells morphology was assessed by incubation of cultures with CellTracker fluorescent dye (Molecular Probes, Eugene, OR, USA) for one hour. The cells were washed with PBS and were fixed in 2% paraformaldehyde for 30 min. Fibroblasts were imaged by fluorescence microscopy. For Phalloidin (Sigma) staining, cells were washed with PBS and were fixed in 2% paraformaldehyde for 30 min followed by Phalloidin staining for 1 h.

### Atomic force microscopy (AFM) imaging

The mechanical properties of cells were measured by AFM using previously established techniques in our laboratory with minor modifications (Qin *et al*., 2014). Briefly, 3D collagen lattices were washed with PBS, and fibroblast morphology was confirmed visually prior to AFM (Fig. 1E). Cell mechanical properties, traction forces and elastic modules/tensile strength, were measured by Dimension Icon AFM system (Bruker‐AXS, Santa Barbara, CA, USA) using PeakForce^™^ Quantitative NanoMechanics mode in fluid wet condition using a silicon AFM probe (PPP‐BSI, force constant 0.01–0.5 N m^−1^, resonant frequency 12–45 kHz, NANOSENSORS^™^, Switzerland). PeakForce^™^ Quantitative Nanomechanical Mapping (QNM^™^) is an AFM technique for measuring nanoscale mechanical properties by calculation of Young's modulus of materials with high spatial resolution and surface sensitivity. AFM was conducted at the Electron Microbeam Analysis Laboratory (EMAL), University of Michigan College of Engineering, and analyzed using nanoscope analysis software (nanoscope analysis v120R1sr3, Bruker‐AXS, Santa Barbara, CA, USA).

### Statistical analysis

Comparisons were made with the paired *t*‐test (two groups) or the repeated measures of ANOVA (more than two groups). Multiple pair‐wise comparisons were made with the Tukey studentized range test. All *P* values are two‐tailed and considered significant when <0.05.

## Author contribution

TQ and GF designed the experiments, analyzed the data, and wrote the manuscript; YS, TH, ZQ, DP, and TQ performed the experiments; JJV discussed the analyses, interpretation, and edited the manuscript.

## Funding

This work was supported by the National Institute of Health (AG19364 to T Quan and G Fisher, AG031452 and AG025186 to G Fisher).

## Conflict of interest

The authors state no conflict of interest.

## Supporting information


**Fig. S1** Reduced cell size/mechanical force inhibits ECM production.
**Fig. S2** Reduced fibroblast size/mechanical force does not change mRNA expression of TGF‐β ligands or Smad2/3/4/7.
**Fig. S3** Specificity of the TβRII adn TβRI antibodies.
