# Supplementary figures and images for: Reduction of fibroblast size/mechanical force down‐regulates TGF‐β type II receptor: implications for human skin aging
Source: Aging Cell. 2015 Oct 8;15(1):67–76. doi: 10.1111/acel.12410 (PMC4717276; doi:10.1111/acel.12410)

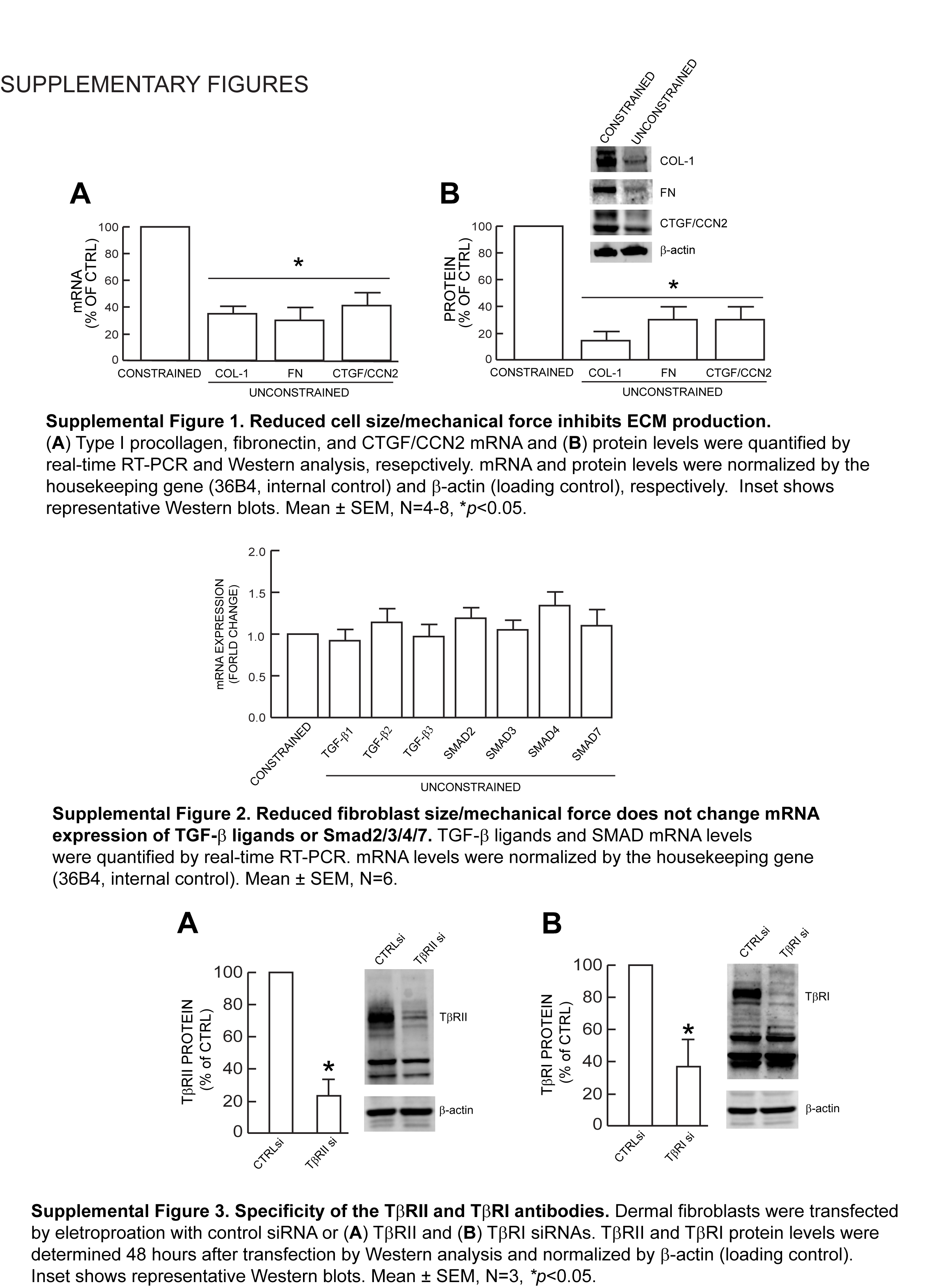

Supplement: Supplementary file 1 — Fig. S1 Reduced cell size/mechanical force inhibits ECM production. Fig. S2 Reduced fibroblast size/mechanical force does not change mRNA expression of TGF‐β ligands or Smad2/3/4/7. Fig. S3 Specificity of the TβRII adn TβRI antibodies. [file ACEL-15-067-s001.tif]
